# An Investigation into the Structure of Wound-Healing Materials, Chemical Materials, Nature-Based Materials, and Wound Monitoring

**DOI:** 10.3390/biomimetics10050270

**Published:** 2025-04-27

**Authors:** HyeRee Han

**Affiliations:** Department of Beauty Art Care, Dongguk University, Seoul 04620, Republic of Korea; luckyherry@hanmail.net; Tel.: +82-10-9130-8087

**Keywords:** wound healing materials, wound healing monitoring, nature-based materials for wound healing, chemical materials for wound healing

## Abstract

With the recent development of advanced industries, in addition to simple abrasions, the demand for wound dressing is gradually increasing in fields such as diabetes care. Factors affecting wound healing include pH, temperature, genetic factors, stress, smoking, and obesity, and studies on these are also increasing. In addition, studies on hydrogels, electrospun nanofibers, foams, films, plant-based materials, chitosan, gelatin, 3D printing, and chemosensors for wound healing are also increasing. However, although there are many data related to wound healing, there are not many studies that have systematically divided them into structures, materials, and monitoring through a review of the literature. Therefore, based on various studies on wound healing, wound-healing materials were classified into structures (films, foams, gauzes, and electrospun nanofibers), chemical materials, nature-based materials, and monitoring sensors, and a literature review was conducted.

## 1. Introduction

Various skin diseases reduce work efficiency and cause an increase in medical costs. Accordingly, research on wound healing is being actively conducted, centering on hydrogels and intelligent monitoring, and when introduced as a clothing material, it is expected to bring about innovation in chronic skin diseases. Factors affecting the wound-healing process include nutrition, bacterial infection, individual health status, age, immunity, stress, smoking, drinking alcohol, wound size, wound depth, wound location, etc.

In addition to simple abrasions, the demand for wound dressing is gradually increasing in fields such as diabetes care, and research on treatment in terms of dressing development, wound monitoring, wound dressing materials, physiological processes (temperature, pH, oxygen levels, and glucose levels), smart dressing, and nanofibers is active [1,2,3,4,5,6,7,8,9,10,11,12,13]. Factors affecting wound healing include local factors such as temperature, oxygenation, and infection, as well as age, diabetes, sexual hormones, genetic components, autoimmune diseases, psychological stress, smoking, and obesity. Therefore, each individual’s rate of wound healing may be different, and appropriate treatment and management are required according to the individual’s condition [14].

In general, wound healing consists of three processes: hemostasis, inflammation, and proliferation remodeling. If there is a defect within these steps, the body’s ability to heal the wound may be hindered. Hemostasis begins immediately after any injury that affects skin integrity. As blood vessels contract, platelets are activated in contact with exposed collagen and release granules, further activating and agglomerating platelets. As a result of platelet activation during hemostasis, a large number of cytokines, including transgenic growth factor-β (TGF-β) and platelet-derived growth factors, are secreted to promote the chemotaxis of neutrophils and macrophages, and the inflammatory phase begins. Within 2 to 3 days of the initial injury, a sufficient number of fibroblasts move to the wound, signaling the onset of the proliferation phase that lasts for up to 3 weeks in the skin wound being healed. Fibroblasts recruited to the wound are converted into myofibroblasts under the influence of several cytokines, which can increase collagen production and eventually reduce the wound [15].

In general, methods for improving biocompatibility include reducing toxicity and improving biodegradability. However, since the body’s immune response and recovery function are very complex, it is not appropriate to talk about the biocompatibility of a single material for a single cell or tissue.

Recently, a relationship between the wound microenvironment and wound healing has gradually been revealed. Therefore, research is being conducted to check the condition of the wound by monitoring the microenvironment in real time. In addition, hydrogels have shown excellent advantages in the field of wound dressing due to their excellent biochemical and mechanical properties. Other previous studies have reviewed the advanced functions of hydrogel dressings, such as their antibacterial activity, attachment and hemostasis, anti-inflammatory and antioxidant activities, substance delivery, self-healing, stimulating response, and conductivity, after summarizing the skin wound-healing process and the associated evaluation parameters [6,16,17]. Another prior study designed a three-layer integrated smart dressing including biomimetic nanofibrous membranes, microenvironmental sensors, and a β-cyclodextrin-containing gelatin methacryloyl (GelMA + β-cd) UV crosslinked hydrogel [16].

The main biological factors that should be evaluated to consider smart substances in vitro and in vivo are antibacterial efficacy, human affinity, and human toxicity. In addition, among chemical and physical factors, the main factors that should be evaluated to consider smart substances in vitro and in vivo are biodegradability, absorbency, tensile strength, bending strength, and abrasion strength.

As such, there are many related data, but there are not many studies that systematically examine these data by dividing them into structures, materials, and monitoring. Accordingly, a variety of studies on wound healing were divided into structures, chemical materials, natural materials, and monitoring.

## 2. Materials and Methods

**Figure 1:**
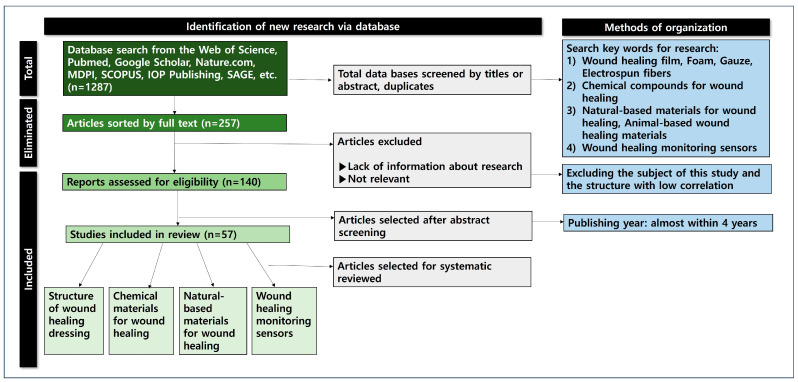
The identification process map of the database research.

This study conducted an in-depth systematic review by classifying wound monitoring and the materials for the introduction of wound-healing clothing materials (Figure 1). Literature research data (*n* = 1287) were collected using databases such as ACS, Harvard.edu, Nature, MDPI, Google Scholar, the Web of Science, IOP Publishing, and PubMed. Most of the documents included were published within the last 4 years (2021–2025). Keywords for the literature research included the following: wound healing film, foam, gauze, electrospun nanofibers, chemical materials for wound healing, natural-based materials for wound healing, wound healing monitoring sensors, etc.

The database list of the included studies was searched for additional eligible publications. The 138 selected papers were divided into four categories (structures, chemical materials, natural materials, and sensors for wound healing) and reviewed in depth. The structures for wound healing were divided into film, foam, gauze, and electrospun nanofibers. The representative materials for wound healing included hydrogels, nanowebs, polyacrylic acid, polyhydroxyethyl methacrylate, and polyacrylamide. In conclusion, this study aimed to provide the foundation for the application of cutting-edge clothing with wound-healing materials and wound monitoring devices.

## 3. Results and Discussion

In this study, the literature on dressing structures, chemical types, natural extract types, and wound monitoring devices for wound healing was considered in depth by category.

### 3.1. Structure of Wound-Healing Dressings

**Figure 2:**
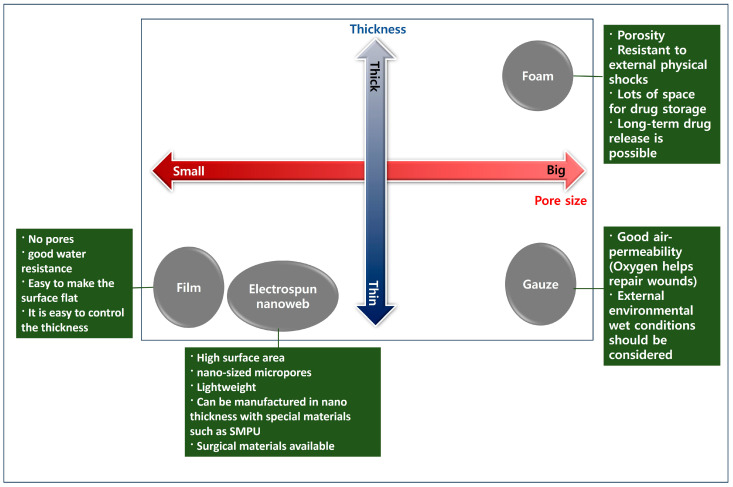
The correlation between wound-healing material structure and characteristics.

According to their structure, different types of wound-healing dressings include film, gauze, foam, hydrocolloid, hydrogel dressings, and electrospun support. Hydrogels are widely used in wound healing due to their similarity to natural extracellular substrates and their ability to provide a humid environment. Multifunctional hydrogels can be prepared with a wide range of properties, including antibacterial, antioxidant, biocompatible, and appropriate mechanical properties. Figure 2 shows the correlation between wound-healing material structure and properties.

#### 3.1.1. Film Dressing for Wound Healing

Films are advantageous because they are flexible and easy to apply, allow for some moisture evaporation, provide a barrier against external contamination, and allow for inspecting the wound floor without removing the dressing [18]. Table 1 shows the systematization of the articles related to film dressing for wound healing.

There are agar/gelatin hydrofilms containing EGF and *Aloe vera* extract, chitosan/PVA-based hydrogel films with honey, ulvan polysaccharides-based hydrogel films, alginate–arginine–zinc ion hydrogel films, carrageenan/galginate/curcuminine hydrogel films, robust carbon dot-based anthraxial CD-PVA films, and polysaccharide-based bioadhesive topical films, and research into these is being extensively conducted [19,20,21,22,23,24,25,26].

**Table 1:** The systematization of articles on film dressing for wound healing, with bibliographic sources.

Authors	Title	Main Content
Melissa Marques Gonçalves., et al.	Preparation and characterization of a novel antimicrobial film dressing for wound healing application [10]	Film dressing, chitosan, Poly(vinyl alcohol), ε-Polylysine, and semi-occlusive dressing
Ioana Savencu., et al.	Review of advances in polymeric wound dressing films [18]	Film, wound dressing, hydrogels, hydrocolloids, hydroactives, foams, alginates, and hydrofibers
H. Xu, F., et al.	Electrospun hierarchical structural films for effective wound healing; [19]	Film, electrospun hierarchical structure, nano- particles, wound healing, antibacterial properties, and cytocompatibility
I. Garcia-Orue., et al.	Agar/gelatin hydro-film containing EGF and *Aloe vera* for effective wound healing [20]	Hydrofilm, agar, gelatin, EGF, *Aloe vera*, and wound healing
H. Chopra., et al.	Preparation and evaluation of chitosan/PVA based hydrogel films loaded with honey for wound healing application [21]	Hydrogel films, honey, chitosan, PVA, and wound-healing application
Jhing-Ee Gan., et al.	Formulation and characterisation of alginate hydrocolloid film dressing loaded with gallic acid for potential chronic wound healing [27]	Film, alginate, pectin, gallic acid, wound dressing, and hydrocolloid film

Melissa Marques Gonc et al. prepared 17 antimicrobial film dressings for wound healing and identified their tensile strength, fracture elongation, Young’s modulus, antimicrobial properties, porosity, water vapor permeable urticaria edema, and cytotoxicity. Twenty-four preparations using the polymer chitosan, poly(vinyl alcohol), and/or ε-polylysine and plasticizer glycerol were designed using a succession design; then, the films were prepared via casting/solvent evaporation. Specifically, the addition of ε-polylysine increased the antimicrobial activity against *Escherichia coli* and *Staphylococcus aureus*. In addition, since it is non-toxic, FD10 is a new film dressing potentially effective for wound-healing applications [10].

Ioana Savencu et al. explained how to make a polymer wound dressing film, including the processing steps. A wound dressing film should be easily removed from the packaging, resistant to breakage, and flexible, with good adhesion to the wound surface and a sufficient capacity to absorb exudate. Finally, it should be able to incorporate active ingredients and release them to the wound site. Polymers used in the manufacture of wound dressing films can have natural or synthetic properties. Natural polymers include chitosan (CS), hyaluronic acid (HA), starch (St), silk fibroin (SF), sericin (Ser), sodium keratin, sodium alginate (SA), gelatin (GE), Janus, and Konzac glucomannan (KGM). Among the synthetic polymers used in the manufacture of wound dressing films, polyvinyl alcohol (PVA), polyacrylic acid (PAA), polycaprolactone (PCL), polyethylene glycol (PEG), and polyvinyl pyrrolidone (PVP) are mentioned. Nanotechnology can be used for wound healing in the form of a drug delivery system that controls the drug release in a wound environment or delivers the drug directly to healing tissues or cells. The benefits are better control of the drug dosage in wound areas, an improvement in drug stability, and the maintenance of a constant drug concentration for a period of time [18].

Haixia Xu et al. a manufactured Janus nanofiber dressing with a hierarchical structure using side-by-side electrospinning technology via eccentric spinners. This hierarchical structure dressing showed excellent antibacterial properties and cell suitability, and the hydrophilic bottom layer provided a good environment for wound healing. In addition, the hydrophobicity of the top layer reduced the adhesion of external microorganisms [19].

Garcia-Orue et al. produced a hydrofilm structure composed of gelatin crosslinked with citric acid, agar, and *Aloe vera* extracts (AV). The addition of epithelial growth factor (EGF) promoted wound healing. Due to the gelatin, the hydrofilm swelled up to 884 ± 36% of the dry weight, which may help control wound moisture. In addition, AV and EGF promoted human keratinocyte and fibroblast migration, and it was argued that these hydrofilms are suitable for chronic wound healing [20].

Hitesh Chopra et al. fabricated a chitosan/polyvinyl alcohol (PVA)-based honey hydrogel film for wound-healing purposes. This hydrogel film was developed via solvent casting, and the water vapor permeability was found to range from 1650.50 ± 35.86 to 2698.65 ± 76.29 g/m^2^/day. The tensile strength and fracture elongation were found to range from 4.74 ± 0.83 to 38.36 ± 5.39 N and 30.58 ± 3.64 to 33.51 ± 2.47 mm, respectively, indicating the mechanical properties of the film. In vitro studies found the continuous release of honey from the film. In addition, the antibacterial performance of the film was investigated for *S. aureus*. Overall, the films produced in this study showed the potential to use chitosan/PVA-based hydrogel films as wound dressings [21].

Jhing-Ee Gan et al. argue that there is an urgent need to develop effective and inexpensive wound dressings for chronic wounds. Hydrocolloid composite films were preformulated by mixing sodium alginate (SA) with various polymer combinations. In this study, all the films loaded with GA were shown to have good wound dressing properties, including the acid pH range (3.97–4.04), moderate viscosity (1600 mPa-s–3198 mPa-s), optimal water vapor permeability (1195 g/m^2^/day, 1237 g/m^2^/day, and 1112 g/m^2^/day), a slow water absorption and film expansion rate, and no chemical interaction between the GA and polymer in the FTIR analysis [27].

As mentioned above, wound-healing films can be produced using various materials (PVA, hydrocolloid, polyacrylic acid (PAA), polycaprolactone (PCL), polyethylene glycol (PEG), citric acid, and *Aloe vera* extract) and can help with advanced wound healing by improving the water vapor permeability and wound recovery speed.

In the case of films, the manufacturing method is relatively convenient, and due to the lack of pores or flat surfaces compared with gauze or nanoweb material, it is more advantageous in terms of external virus protection and waterproofing than other structures. In the case of a film, the pore size is 0 nm, and when the electrospinning nanoweb thickness (nonstretch state) is 20 to 60 μm, the pore size is about 272 to 473 nm [28]. Therefore, since the film does not have pores, the effect of blocking the inflow of viruses is better than that of the nanoweb or gauze.

Therefore, it is believed that the film method will be advantageous when used in an environment with a lot of moisture.

#### 3.1.2. Foam Dressing for Wound Healing

Amit Gefen et al. argued that the mechanical performance (friction properties, suitability, swelling properties, and durability) of foam materials is highly dependent on the microstructure of the foam components, especially the microtopography, density, and porosity [29]. Table 2 shows the systematization of articles on foam dressing for wound healing.

Studies on foam dressings for wound healing include polyethylene glycol/triethoxysilane-modified polyurethane foam dressings, silver-releasing foam dressings for diabetic foot ulcer healing, alginate foam gels modified using graphene oxide, controlled-release iodine foam dressings, shape memory polymer foams, silver-based foams, and polyurethane/Ag composite foams [29,30,31,32,33,34,35,36,37,38,39,40,41,42].

Chiu Fang Chen et al. attempted to promote wound healing by preparing a superabsorbent polyurethane (PU) foam dressing modified with polyethylene glycol (PEG) and triethoxysilane (APTES). The PEG-modified (PUE) and PEG/APTES-modified (PUESi) dressings were prepared via self-foaming reactions. The PUESi dressing shortened the inflammatory stage and enhanced collagen accumulation. In conclusion, the PUESi dressing reinforced wound healing by generating micro-negative pressure with a simple preparation method and high absorbency according to the deformation [30].

Yu-Chi Wang et al. investigated the wound-healing efficacy of silver-releasing foam dressings and silver-containing creams in managing outpatients with diabetic foot ulcers (DFUs). The treatment group received silver-releasing foam dressings (Biatain^®^ Ag Non-Adhesive foam dressings; Coloplast, Humlebak, Denmark). The control group received 1% silver sulfadiazine (SSD) cream. The ulcer area in the silver foam group was significantly reduced after 4 weeks of treatment compared with the SSD group (silver foam group: 76.43 ± 7.41%; SSD group: 27.00 ± 4.95%, *p* < 0.001). It was argued that the silver-releasing foam in this study could be an effective wound dressing for DFU primarily at the beginning of wound management [31].

Cunren Chen et al. explored the mechanisms involved in diabetic wound healing during the treatment of foam dressings (FDs) or micropower vacuum dressings (MVDs). This study found that wound healing in mice with DFUs was improved upon application of a FD and MVD. The therapeutic efficacy of the FD was superior to that of the MVD. Compared with the diabetic onset group, the concentrations of inflammatory cytokines, tumor necrosis factor alpha, interleukin-1β, and interleukin-6 were significantly downregulated [32].

The foam gel dressing produced by Feng Xie et al. consisted of a chitooligosaccharide-modified graphene oxide (CG) nanocomposite and calcium alginate foam substrate. In this system, the CG had strong interaction with platelets, which aided in rapid hemostasis. Thus, the wound dressing quickly stopped bleeding within 10 s. Whole-layer wound-healing experiments showed that when compared with a blank control and CG-free foam gel dressing, the CG-loaded foam gel dressing exhibited better healing properties, and the wound covered with it almost completely healed within 12 days [33].

Kevin Woo et al. studied patients’ reported experiences, foam dressings, the pain, wound exudate, unpleasant smell, itchiness, and self-management needs. The fluid absorbent of the dressing and the holding core integrated multiple layers. In addition, the fluid could be fixed without leaking back to the wound or skin. It also facilitated timely fluid movement (vertical) from the wound [34].

Watson et al. evaluated the antimicrobial properties of commercially available wound dressings with different material compositions and antimicrobial agents against several in vitro microbial and biofilm models. The continuously released iodine foam and silver-impregnated carboxymethylcellulose (CMC) wound dressing materials showed strong biofilm management properties compared with a foam dressing containing methylene blue and gentian violet. In addition, although both silver- and iodine-containing materials reduced *S. aureus* to the detection limit, *P. aeruginosa* growth was completely reduced only by the continuously released iodine foam dressing materials [35].

Changling Du et al. incorporated phenolic acid (PA) into the network of shape memory polymer (SMP) polyurethane foam by reacting it with isocyanate. They also characterized the shape memory properties, antimicrobial and antioxidant activities, and blood and cell interactions of SMP foam with PA. Empty inserts were used as positive controls (*n* = 3), 30% H_2_O_2_ was added to the negative controls, and silver-based foam dressings (AREZA Medical, Tx, Dallas) were used as clinical controls. The SMP foam with PA maintained the antimicrobial and antioxidant properties of integrated PA and exhibited good antimicrobial properties against approximately 20% H_2_O_2_ erasure and *E. coli* (approximately 5-fold reduction in CFU compared with the control foam), *S. aureus* (approximately 4.5-fold reduction in CFU compared with the control foam; the number of CFU was similar to the clinical control), and *S. epidermidis* (approximately 25–120-fold reduction in CFU compared with the control foam; the number of CFU was similar to the clinical control) [36].

As mentioned above, wound-healing foam can be made using various materials (polyurethane foam dressing, silver-releasing foam pressing, and alkaline foam gel modified using graphene oxide) and can improve wound healing due to its characteristics of porosity, light weight, shape memory, and antibacterial properties.

The foam structure has the advantage of being stronger against external physical shocks because it is bulky than films, gauze, and nanowebs. In addition, the foam structure has the advantage of being advantageous for long-term drug release due to a large amount of drug absorption space. Accordingly, when there is a large external impact or wound-healing area, the foam structure is considered to be appropriate.

#### 3.1.3. Gauze for Wound Healing

Studies on wound-healing gauze include layer-by-layer coating of carboxymethyl chitosan–gelatin–alginate on cotton gauze, bimetallic silver–platinum (AgPt) nanoparticles and chitosan-fabricated cotton gauze, cotton gauze with gallic acid, gallocatechin–silver nanoparticle-impregnated cotton gauze, calcium–copper zeolite gauze, freeze–thawing chitosan/ions hydrogel-coated gauzes, etc. [43,44,45,46,47,48,49,50,51,52,53,54,55,56,57,58].

Table 3 shows the systematization of articles on gauze dressings for wound healing.

Liang et al. incorporated polylysine, a natural antibacterial peptide, into traditional cotton fiber dressing to produce a wound dressing with favorable antibacterial properties and biosafety. They argued that the cotton fiber dressing provides excellent moisture absorption and softness, while polylysine provides excellent biocompatibility, an extensive antibacterial spectrum, and high stability [43].

Qian et al. performed a meta-analysis of 10 randomized controlled trials (RCTs) in 2780 patients. Patients in the negative pressure guard therapy (NPWT) group had a lower overall infection rate (MD = 0.70, 95% CI: 0.54–0.90, *p* = 0.005), lower acute wound infection rate (MD = 0.35, 95% CI: 0.16–0.77, *p* = 0.009), and shorter hospital stay (MD = 24.00, 95% CI: 6.82–84.46, *p* < 0.001). The NPWT group had a higher proportion of patients recovered from wounds than the control group [40].

After split skin was collected, Liu et al. divided the donation site into two parts along the median line. BHBO gauze was applied to one-half of the donation wounds, and Vaseline gauze was applied to the other half [45]. The wound-healing time, pain scores on postoperative days 3, 6, and 9 and the Vancouver Scar Scale (VSS) scores at the 6-month follow-up were evaluated. The wound-healing time was significantly shorter in the Ba-Hao burn point (BHBO) group than in the control group (10.07 ± 1.48 days vs. 11.50 ± 1.74 days, *p* < 0.001). The pain scores quantified using visual analog scores on postoperative days 3 and 6 were significantly lower in the BHBO group than in the control group (5.33 ± 1.54 and 4.17 ± 1.51 vs. 7.57 ± 1.41 and 5.20 ± 1.47, respectively). The BHBO showed faster donation site healing, decreased postoperative pain, and improved scar quality in the 6-month follow-up compared with the case with petroleum jelly gauze alone.

Fang et al. developed a reusable ionic liquid–solid antimicrobial cotton gauze wound dressing using 1-vinyl-3-butylimidazolium bromide, 3-aminopropyltriethoxysilane, glycidyl methacrylate, and butyl acrylate [46]. The antimicrobial cotton gauze showed effective antimicrobial ability against *E. coli*, *S. aureus*, and *C. albicans* and did not show cytotoxicity to L929 mouse fibroblasts.

Yang et al. developed a bifunctional CG dressing (CPCG) by chemically grafting polyhexamethylene guanidine (PHMG) and physically adsorbing chitosan (CS) onto a cotton gauze (CG) surface [47]. Due to the strong bactericidal activity of HMG, CPCG exhibited excellent immediate and sustained antibacterial activity against Gram-positive and Gram-negative bacteria. In addition, CS’s abundant hydroxyl and amino groups conferred superior biocompatibility, moisture absorption, moisturizing, and cell scratch healing performance on the CPCG.

Nagarjuna Reddy et al. examined the wound-healing ability of gallocatechin (GC)- and silver nanoparticle (AgNP)-impregnated patches in diabetic mice [48]. The GC-AgNPs-CGP (CGP2 and CGP3) dressing on diabetic wound mice decreased the change in the Wnt3a/β-catenin pathway, resulting in decreased cell death and increased proliferation, which significantly improved the diabetic wound healing.

Xiao et al. developed a simple freeze–thaw strategy in which multiple ions are released on demand to accelerate wound healing by incorporating chitosan/ion hydrogels into medical gauze. In vitro studies showed that the gauze can temporarily release multiple metal ions on demand, and the released metal ions are effective in killing bacteria and promoting cell migration. Further histological analysis showed that these metal ion-loaded gauzes accelerated wound healing by promoting granulation formation, collagen accumulation and maturation, re-epithelialization, and vasogenesis and by regulating the expression of inflammatory factors (e.g., tumor necrosis factor-α) and the polarization of macrophages to inhibit inflammation [49].

As mentioned above, wound-healing gauze can be manufactured using various materials (layer-by-layer coating of carbonoxymethyl chitosan–gelatin–alginate on cotton gauze, bimetallic silver–platinum (AgPt) nanoparticles, and chitosan-fabricated cotton gauze) and can positively affect wound-healing improvement due to its excellent air permeability, porosity, cell migration promotion, and sterilization activity.

Gauze has the advantage that a large amount of oxygen can be introduced into the wound due to its excellent porosity. However, due to its low waterproofing property, it is necessary to consider the wet state of the external environment when used in the wound. Since oxygen greatly helps in wound recovery, the use of gauze is recommended over foam and film in an environment where there is no effect of external moisture.

#### 3.1.4. Electrospun Nanofibers for Wound Healing

Electrospun nanofibers are produced by applying a high voltage to a cylinder containing a polymer material so that the nanofibers collect in the cylinder to form a Taylor cone. Electrospun nanofibers have a high surface area and can be used for wound healing, surgical sutures, moisture-permeable waterproof materials, wearable computers, etc. [28].

Due to the large surface area structure of nanofibers, more drugs can be contained, which can be effectively delivered to the wound site. Table 4 shows the systematization of articles on electrospun nanofibers for wound healing.

Studies on electrospun nanofibers for wound healing include chitosan nanofibers, sandwich-type nanofibers with curcumin, diabetes healing, polysaccharides-mediated electrospun nanofibers for diabetic patients, PVA@ PLA electrospun nanofibers embedded with Bletilla striata polysaccharide, etc. [59,60,61,62,63,64,65,66,67,68,69,70,71,72,73,74,75,76,77,78,79].

Bao et al. prepared hyaluronic acid (HA)/graphene (Gr) electrospun fiber films containing polyphenolic tannic acid (TA). The HA/Gr/TA electrospun fiber films with a TA concentration of 0.3% *w*/*v* showed the best antioxidant activity and better mechanical, moisture absorption, moisture retention, and degradation properties than films without TA. In addition, they showed superior antimicrobial activity and biocompatibility to the films without TA and promoted healing in infected wounds [59].

Xu et al. prepared electrospun polyvinyl alcohol (PVA) and bone marrow-derived stem cells (BMSCs) with handheld electrospinning devices; the distribution and interaction of the cells and fibers were determined using optical and electron microscopy, and the cell viability and proliferation were determined using live cell/dead cell staining. The manufactured electrospun material strongly promoted full-layer skin wound repair in rats. The proposed electrospun technique is expected to have great potential for home, outdoor, and full-length first aid [60].

Liu et al. argued that a physiologically active nanofiber dressing produced by electrospinning technology considers the characteristics of the wound-healing process, stimulates cell migration, and regulates inflammation. At the same time, it relieves pain by releasing related drugs and can be effectively used to treat acute or chronic wounds. However, this advanced dressing should be evaluated in a large number of clinical trials before final clinical application [61].

Guo et al. argued that due to the current increase in clinical requirements, the application of vegetable materials to nanotechnology is emerging, which is generally fused with electrospinning, to form nanofiber membranes suitable for skin wound healing. They argued that the continuous screening of herbs and their derivatives suitable for wound dressing in complex clinical situations is particularly important, because wound-healing processes involve the coordination of various cells and molecules in clinical practice [62].

Designed by Chen et al. for wound healing, gel nanofiber materials can synergize the benefits of hydrogel and nanofibers to overcome the bottleneck of hydrogel’s low mechanical strength and self-adhesion, as well as the lack of a healing environment due to nanofibers. A nanofiber scaffold composed of polycaprolactone/poly(citric acid)-ε-lysine (PCL/PCE) nanofibers was manufactured through a novel strategy of microfluidic electrospinning, which provided a basis for hyaluronic acid–polylysine (HE) gel growth in nanofibers [63].

Su et al. determined that electrospun fibers containing hyaluronic acid (HA) and keratin (KR) are promising for wound dressing applications. In vitro studies showed that the spinning mat had no cytotoxic effect, and the HA and KR incorporated into the fiber structure synergistically increased the cell viability and cell proliferation [64].

As mentioned above, wound-healing electrospinning nanofibers can be produced using various materials (shape memory polyurethane, polysaccharide PVA@PLA, hyaluronic acid with polyphenol tannic acid (TA), chitosan, etc.) and can have a positive effect on improving wound healing due to their large surface area, light weight, air permeability, ability to overcome bottlenecks, cell senescence, and increased cell proliferation.

The electrospinning nanoweb has the advantage of having nano-sized micropores and being very light, with a thickness of nanometers to micrometers. There is also an advantage in that the pore size changes according to the external temperature when the electrospinning nanoweb is manufactured with shape memory polyurethane. This has the advantage that it can be applied to surgical sutures.

### 3.2. Chemical Materials for Wound Healing

Zhao et al. argued that the use of functional materials to promote wound healing is receiving considerable attention [80]. Table 5 shows the systematization of articles on chemical materials for wound healing.

Chemical materials for wound healing include hydrogel films composed of acrylic polymers, polyethylene, and phenoxyethanol; shape memory materials; mechanically strong hydrogels containing polyethylene glycol and polyvinylpyrrolidone; and dressings containing silver and iodine. Drug delivery technology using hydrogel has the advantage of being able to slowly release drugs to specific areas. There is also research on wound-healing materials with improved antibacterial effects among these compounds. Chemicals for wound-healing materials include silver, copper, immunomodulatory hydrogels, ODHTCC-pADM(OD-pA), etc.

Studies on chemical materials for wound healing include hemostatic materials, antibacterial electrospun nanofiber materials, arginine-based materials, nitric oxide-releasing biomaterials, metal–organic structures, polyacrylamide/chitosan hydrogels, conductive materials, metal–organic skeleton-based dressings, and copper-based materials [3,12,80,81,82,83,84,85,86,87,88,89,90,91,92,93,94,95,96,97,98,99].

Fu et al. explained that nanomaterials have unique physicochemical properties and functions and have been widely applied in wound treatment. In addition, the nanomaterials’ size, shape, and chemical properties can be precisely controlled, which is effective in improving drug solubility, stability, targeting, antibacterial, and wound-healing ability and provides better adaptation to complex and diverse wound environments [81].

Gowda et al. argued that nanoparticles (NPs) used for wound healing are mainly classified into three types: inorganic/metallic NPs, lipid-based NPs, and polymeric NPs. Several research reports have demonstrated that polymeric and lipid nanoparticle drug delivery systems accelerate the wound-healing process due to their ability to prevent therapeutic agents from being degraded by specific wound environments (pH, temperature, enzymes, etc.). In addition, the loaded therapeutic agents were slowly and stably released from these nanomaterials, maintaining an effective treatment concentration at the wound site, reducing the frequency of administration, and accelerating the wound-healing process [82].

Diao et al. argued that copper is mostly found in the body in the same binding form to form copper proteins. It also argued that copper can improve the body’s specific and non-specific immune functions, and it may also act as an antibacterial agent by producing active oxygen species (ROS) and photothermal effects against antibiotic-resistant bacteria [83].

Kharaziha et al. aimed to reproduce chronic wounds via the configuration of skin tissue with the functions of chronic wounds. In addition, a strategy to adjust the immune response is important to ensure high-quality cells. For example, hydrogels can be used to prevent cell death by encapsulating different types of immune cells and delivering cytokines. Eventually, they can recruit host immune cells and induce a superior immune response [84].

Huang et al. aimed to integrate oxidized chitosan 24th (ODHTCC) salt into a porcine acellular thermal matrix (pADM) to create an antibacterial collagen scaffold dressing utilizing the Schiff base reaction. The OD-pA (ODHTCC-pADM) exhibited excellent antibacterial properties, showing inhibition rates of 95.6% and 99.9% against *E. coli* and *Staphylococcus aureus*, respectively, representing cytotoxicity level 1, thus meeting the in vitro requirements of national biomedical materials [85].

Cheng et al. extracted carbon dots (CDs) from resveratrol, a natural plant compound, and used citric acid to improve the water solubility. In a mouse skin defect model, resveratrol-based carbon dots (RES-CDs) promoted wound healing and stimulated vascularization and tissue regeneration near the wound site, which was demonstrated by the increased CD31 and VEGF expressions. Resveratrol-derived CDs with enhanced water solubility exhibited superior performance in tissue healing compared with resveratrol [86].

A method of controlling the porosity of the material using siloxane–acrylate latex and carbon fiber as pore-forming templates has been proposed by Papynov et al. And histological analysis and multi-sliced CT confirmed the ability of the biomaterial to bind to the mandibular defect (alveolar tree) of the test animals. Blood count tests showed that these implants did not have a direct toxic effect on living organisms [87].

As explained above, substances used for wound healing include hydrogel films composed of polyethylene and phenoxyethanol, shape memory materials, mechanically strong hydrogels including polyethylene glycol and polyvinylpyrrolidone, silver, and copper, and among these substances, there were many cases that positively affected wound healing with antibacterial properties, tissue regeneration stimulation, and blood vessel formation. Therefore, these substances have shown applicability in mass production for wound-healing industries.

### 3.3. Natural Materials for Wound Healing

El-Ashram and others explained that natural-product-based wound-healing treatments include plant-derived natural products, insect-derived natural products, and marine-derived natural products [14]. Table 6 shows the systematization of articles on nature-based wound-healing materials.

In terms of wound-healing materials, natural extracts include *Aloe vera* leaves, southern African medicinal plants, hypericum hookerianum, *Hibiscus sabdarifa calyx* extract, *Moringa olei pera* leaf extract, and spirulina platensis extract. It is argued that natural extracts have an advantage in biocompatibility. In addition, nature-based wound-healing studies address biomaterials (gelatin, collagen, silk sericin, and chitosan), nature-based bioink, antioxidant biochemistry, etc. [14,100,101,102,103,104,105,106,107,108,109,110,111,112,113].

**Table 6:** The systematization of articles on nature-based materials for wound healing, with bibliographic sources.

Authors	Title	Main Content
El-Ashram, S., et al.	Naturally-derived targeted therapy for wound healing: Beyond classical strategies [14]	Naturally derived, wound healing, hemostasis, inflammatory, plant-derived products, insect-derived products, and marine-derived products
Yang, W., et al.	Biomimetic natural biopolymer-based wet-tissue adhesive for tough adhesion, seamless sealed, emergency/nonpressing hemostasis, and promoted wound healing [111]	Biomimetic natural biopolymer, wound healing, wet-tissue adhesive, adhesion, seamless sealed, and emergency
Masri, S. and M.B. Fauzi	Current insight of printability quality improvement strategies in natural-based bioinks for skin regeneration and wound healing [112]	Nature-based bioinks, 3D bioprinting, skin regeneration, wound healing, and physicochemical and mechanical properties
Naomi, R., et al.	Natural-based biomaterial for skin wound healing (Gelatin vs. collagen): Expert review [113]	Nature-based biomaterial, gelatin, collagen, and skin wound healing
Nichcha Nitthikan., et al.	Exploring the Wound Healing Potential of a *Cuscuta chinensis* Extract-Loaded Nanoemulsion-Based Gel [114]	*Cuscuta chinensis*, nanoemulsions, wound care, anti-inflammation, and molecular docking
Shivani Dogra., et al.	Phytochemical Analysis, Antimicrobial Screening and In Vitro Pharmacological Activity of Artemisia vestita Leaf Extract [115]	Artemisia vestita, antimicrobial, cytotoxicity, anti-inflammatory, antioxidant, and wound healing
Maite Rodríguez-Díaz., et al.	Antimicrobial Activity and Phytochemical Characterization of Baccharis concava Pers., a Native Plant of the Central Chilean Coast [116]	Baccharis concave, antimicrobial activity, flavonoids, caffeoylquinic acid, and phenolic compounds
Cao, X., et al.	Animal tissue-derived biomaterials for promoting wound healing [117]	Animal tissue, biomaterials for promoting wound healing, extracellular matrix (ECM), collagen, and chitosan
Napavichayanun, S., et al.	Effect of animal products and extracts on wound healing promotion in topical applications: a review [118]	Animal extracts, chitosan, collagen, honey, protein-aided steroids, silk sericin, peptides, anti-inflammatory, antibacterial activity, moisturizing effect, and biocompatibility
Da, L. C., et al.	Progress in development of bioderived materials for dermal wound healing [119]	Amniotic membrane (AAM), collagen, elastin, laminin, fibronectin, and small intestinal submucosa (SIS)
Chouhan, D., et al.	Trends in bio-derived biomaterials in tissue engineering [120]	Bio-derived biomaterials in tissue engineering, collagen, gelatin, and fibrin
Esmaeili, A., et al.	Acellular fish skin for wound healing [121]	Acellular fish skin, 3D cell-free support for skin regeneration, and extracellular matrix content
Zheng, F., et al.	Host response after reconstruction of abdominal wall defects with porcine dermal collagen in a rat model [122]	Porcine skin collagen, Pelvi-col™, Prolene™, and collagen accumulation
Baldursson, B. T., et al.	Healing rate and autoimmune safety of full-thickness wounds treated with fish skin acellular dermal matrix versus porcine small-intestine submucosa: a noninferiority study [123]	Fish skin ADM, porcine small-intestine submucosa, and pig small submucosal extracellular matrix

Yang et al. designed robust wet tissue adhesives based on collagen and starch materials (CoSt). The new bioadhesive showed repeatable strong wet tissue adhesion (62 ± 4.8 KPa), high sealing performance (153.2 ± 35.1 mmHg), rapid self-healing ability, excellent injectability, and morphological adaptability [111].

Masri et al. explained that several biomaterials derived from nature-based bioinks (combining biomaterial inks for 3D bioprinting) such as collagen, gelatin, alginate, fibrin, hyaluronic acid (HA), chitosan, and agarose, have become the preferred bioinks for fabricating bioscaffolds. Most nature-based bioinks have excellent biocompatibility, a rapid biodegradation rate, non-toxicity, and optimal mechanical stability [112].

Naomi et al. argued that both Col (Collagen) and gelatin are natural biomaterials, which are widely used as therapeutic agents, especially for healing skin wounds. As natural Col has no antimicrobial properties, incorporating antimicrobial effects into Col further improves its physicochemical properties. Col can be incorporated with silver nanoparticles (AgNP), collagenin, epoxidized saprol, polyhexamethylene biguanide, titanium dioxide, and more. In addition, natural gelatin does not exhibit antimicrobial effects either. However, gelatin can easily be incorporated with ginkgo extract, D-limonene, pinibox, and black pepper oleoresin [113].

Nitthikan et al. evaluated the biological activity, including antioxidant, antimicrobial, and anti-inflammatory activities, and toxicity of *C*. *chinensis* seed extract. The ethyl acetate *C*. *chinensis* seed extract showed better metal-reduction properties, lipid peroxidation inhibition, and antimicrobial activity than the ethanol *C*. *chinensis* seed extract [114].

Dogra et al. investigated the plant-based components, antimicrobial activity, and antioxidant, anti-inflammatory, cytotoxic, and wound-healing potentials of *A. vestita* leaf extract (ALE). The results showed 94.6% wound obstruction (after 24 h of incubation) compared with the positive control cipladin, as shown with *Staphylococcus aureus* (14.2 ± 0.28 mm), *Escherichia coli* (17.6 ± 0.52 mm), *Bacillus subtilis* (13.1 ± 0.37 mm), *Streptococcus pyogenes* (17.3 ± 0.64 mm), Proteus mirabilis (9.4 ± 0.56 mm), *Aspergillus niger* (12.7 ± 0.53 mm), *Aspergillus flavus* (15.3 ± 0.25 mm), and *Candida albicans* (17.6 ± 0.11 mm). In addition, scratch analysis results showed 94.6% wound obstruction (after 24 h of incubation), which evidenced remarkable wound-healing activity compared with the positive control cipladin [115].

Rodríguez-Díaz et al. described the phytochemical composition and antimicrobial properties of *Baccharis concava Pers*. (sin. *B. macraei*), a shrub found near the Pacific coast. Studies have shown that *B. concava* extracts have strong antimicrobial activity, possibly due to the presence of metabolites derived from phenolic acids such as caffeoylquinic acid and flavonoids such as quercetin, which have been argued to be helpful in wound healing [116].

Xinyue Cao et al. studied wound-healing promotion using animal tissue-derived biomaterials, collagen, chitosan, extracellular matrix (ECM), and silk fibrin. They also argued that animal tissue-derived biomaterials have emerged as promising candidates for wound healing due to their abundant sources, low side-effect profiles, excellent biological activity, biocompatibility, and unique extracellular matrix (ECM) mimicry [117].

Supamas Napavichayanun et al. described various kinds of animal-derived products, including chitosan, collagen, honey, protein-aided steroids, silk sericin, peptides, and proteoglycans, in terms of their mechanisms of action, pros, and cons, when applied as wound-healing promoters. The benefits of these animal products are wound-healing promotion, anti-inflammatory and antibacterial activities, moisturizing effect, biocompatibility, and safety. However, disadvantages such as allergies, low stability, interbatch variability, and high extraction and purification costs were inevitable in some products [118].

Lin-Cui Da et al. explained that the cellular amniotic membrane (AAM), which consists of collagen, elastin, laminin, fibronectin, and growth factors, is an attractive biomaterial for use in wound healing because it reduces pain and wound dehydration, promotes epithelialization, prevents scar formation, and exhibits anti-inflammatory, antibacterial, and anti-fibroblast effects. In addition, small intestinal submucosa (SIS), an ECM (extracellular matrix) material derived from pigs, is more advantageous in promoting angiogenesis, cell growth and differentiation, and tissue regeneration [119].

Animal-derived biomaterials, such as collagen, gelatin, fibrin, and hyaluronic acid, by Dimple Chouhan et al., have greatly contributed to the success of tissue engineering so far. These materials are often preferred over synthetic counterparts due to their physiological relevance, unique cell–material interactions, and biocompatibility [120].

Ali Esmaeili et al. argued that fish skin could be introduced as a potential alternative to other grafts. Through a proper cell removal process, it is possible to design a 3D cell-free support for skin regeneration without compromising its shape and extracellular matrix content. Therefore, the role of the cell removal process is very important in maintaining the properties of the fish skin. It was explained that the manufacturing process includes chemical, physical, and enzymatic methods [121].

Fang Zheng et al. studied the host response after the reconstruction of peritoneal defects using porcine skin collagen in a rat model. A whole-layer peritoneal defect was created in 64 Wistar rats and reconstructed with Pelvicol™ or Prolene™. The animals were evaluated for changes in hernias, infection, adhesion, and the tensile strength of the implants and at sacrifice on days 7, 14, 30, and 90. Pelvicol causes a milder inflammatory response than Prolene, exhibits less adhesion formation and more orderly collagen accumulation, and only reaches similar tensile strength results after 90 days [122].

Using a non-inferiority test, Baldur Tumi Baldursson and others compared the effects of fish skin ADM to pig small submucosal extracellular matrix in the healing of 162 full-layer 4 mm wounds on the forearms of 81 volunteers. As a result, fish skin products were non-inferiorated at the primary endpoint and healed in 28 days. Moreover, wounds treated with fish skin cell-free substrates healed considerably faster [123].

As explained above, nature-based materials used for wound healing include *Aloe vera* leaves, southern African medicinal plants, *Hypericum hookerianum*, *Hibiscus sabdaripa calyx* extract, gelatin, collagen, silk sericin, nature-based bioink, antioxidant biochemistry, pig skin, fish, and chitosan, and among these substances, there are cases that positively affect wound healing via anti-inflammatory properties, antioxidants, non-toxicity, and rapid self-healing ability, showing potential for application in the wound-healing industry. Natural materials for wound healing are diverse, including aloe, gelatin, *Cuscuta chinensis*, collagen, and chitosan, as described in the paper. However, it is judged that the effectiveness of these substances depends on race, age, wound area, wound depth, smoking, habits, and immunity.

### 3.4. Wound-Healing Monitoring Sensors

Wound monitoring devices have been reported to help reduce expensive laboratory tests associated with long-term hospitalization, multiple doctor visits, and chronic wound diagnosis and treatment. Indicators specific for chronic wounds include pH, temperature, oxygen, blood pressure, lactate, glucose, and infection status. The sharper the sensor detection, the more accurate the wound recovery can be detected, which can effectively affect the quick perfect wound recovery.

Research on wound-healing monitoring sensors includes multimodal detection and treatment systems, wearable sensors and systems for pH and temperature detection, wearable electronics, artificial intelligence chronic skin monitoring, corn protein electrospun fibers, non-contact monitoring wearable sensors, IOT-based smart wound sensors, fiber-based protein sensors, etc. [124,125,126,127,128,129,130,131,132,133,134,135,136,137,138,139,140,141]. Table 7 shows the systematization of articles on monitoring wound healing.

Yuheng Zhang et al. recently argued that the relationship between the wound microenvironment and wound healing has become increasingly clear, and thus, the wound condition can be roughly confirmed by monitoring the microenvironment in real time. They built a microenvironment sensor, comprising a sensing chip and a control module, encapsulated in a white plastic case. The sensing chip was a printed circuit board welded with temperature and humidity sensors (HDC1080DMBR, Texas Instruments, Dallas, TX, USA) and air pressure sensors (LPS22HBTR, STMicroelectronics, Geneva, Switzerland). This study demonstrated the function of an integrated smart dressing with biomimetic nanofiber membranes, microenvironment sensors, and GelMA + β-cd UV crosslinked hydrogels to monitor the wound microenvironment and promote angiogenesis and wound healing. Specifically, they argued that hydrogels can significantly accelerate neovascularization, granule tissue remodeling, and epithelial expansion in vivo by increasing the expression of HIF-1α, and the sensors showed excellent measurement accuracy, stability, biological durability, and biocompatibility [16].

Youn et al. explained that an electronic skin (E-skin) is an electrical device or system that mimics the mechanical elasticity, adaptability, self-healing, and biocompatibility of the skin, with advanced sensory functions through mechanical receptors. With recent advances in the science of flexible electronic devices and soft materials, E-skins for wearable health monitoring, medical devices and implants, artificial prosthetic skin, humanoid robots, and human–machine interface applications are rapidly being developed [142].

Wang et al. researched the design principles, performance characteristics, and self-driving applications of hydrogel-based energy harvesters that utilize the core technologies of triboelectric nanogenerators (TENGs), piezoelectric nanogenerators (PENGs), and thermoelectric generators (TEGs). The piezoelectric effect and triboelectric electrical effect are commonly used mechanisms for human motion detection. In addition, although hydrogels are suitable for wearable applications due to their excellent flexibility and adaptability, most of the research on hydrogel sensors has been conducted with wired connectivity [143].

Wu et al. showed that recent advances in three-dimensional (3D) printing and real-time monitoring technology have made it possible to create tissue-like membranes and provide appropriate microenvironments. Three-dimensional membrane-binding sensors can help monitor and control wound infections. Traditional sensors for regenerative skin membranes include pH-sensitive 3D membranes, temperature sensors, oxygen-sensitive membranes, protein-sensitive membranes, and metabolic disease sensors. In addition, sensors are used for other applications, including blood pressure, and 3D membrane-binding sensors also play an important role in the regeneration of the heart, blood vessels, and bones [144].

Through 3D printing, a drug delivery function can be put into the scaffold to see the wound-healing effect. In addition, a new 3D-printed scaffold designed to improve bone healing by improving blood vessel formation can be produced. In this case, polylactic acid- and calcium phosphate-based glass are combined to create a structure that supports blood vessel formation during bone regeneration.

Jin et al. proposed hydrogel dressings that monitor wound conditions and provide customized treatment, such as pH, temperature, glucose, and pressure sensitivity, as well as nanocomposite hydrogel dressings. In recent years, researchers have obtained nanocomposite hydrogel dressings by introducing nanoparticles or nanorods into the hydrogel network. Multifunctional hydrogel dressings can monitor the wound microenvironment and can also have superior antibacterial, anti-inflammatory, anti-bleeding, mechanical, injectable, and self-healing properties [145].

Wang et al. argued that hydrogels have antibacterial, hemostatic, and adhesive properties. A personalized wound management model with high accuracy (94.47%) based on convolutional natural network (CNN) machine learning algorithms can analyze and evaluate wound healing and infection status through colorimetric signals from hydrogel dressings [17].

As mentioned above, TENGs (triboelectric nanogenerators), PENGs (piezoelectric nanogenerators), TEGs (thermoelectric generators), and 3D printing can be used for wound-healing monitoring, and these have a positive effect on wound healing, as they can monitor the microenvironment of the wound; analyze and evaluate the pH, temperature, wound healing and infection status; and detect oxygen and protein. This suggests the possibility of applications to the future wound-healing monitoring industry, including chemosensors and wearable computers.

**Figure 3:**
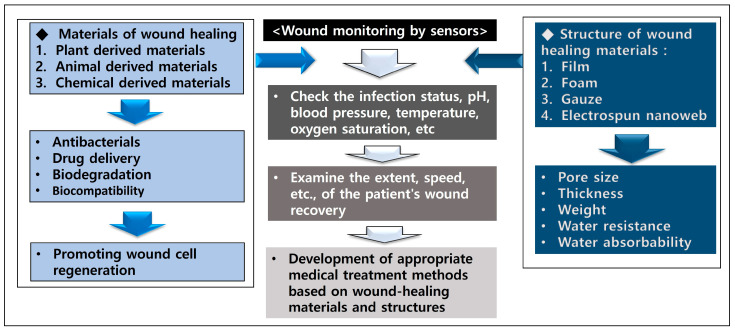
Correlations regarding wound-healing materials, structures, and monitoring.

The correlations regarding wound-healing materials, structures, and monitoring are shown in Figure 3. As shown in Section 3.2 and Section 3.3 above, wound-healing materials have a positive effect on antimicrobial activities at the wound and the promotion of cell regeneration. In addition, wound monitoring is expected to have a positive effect on the development of appropriate medical treatment methods according to wound-healing materials by checking the patient’s wound recovery rate, pH, temperature, oxygen, and infection status.

## 4. Conclusions

Through the results of this study, we described wound-healing materials, their structures, chemical and natural materials, and monitoring mechanisms.

The classification according to the structure of wound-healing dressings includes electrospun nanofibers, films, gauze, foam, hydrocolloids, hydrogels, etc.

- Current status

Antimicrobial hydrogel wound dressings are expected to remove bacteria and accelerate the healing process. Hydrogels are widely used in wound-healing fields due to their similarity to natural extracellular substrates, their ability to provide a wet environment, and their adhesive properties. Hydrogels such as gelatin bases also exhibit a macroporous structure, excellent biocompatibility, and excellent anti-inflammatory effects. In addition, multifunctional hydrogels can be prepared with a wide range of properties, including antibacterial, antioxidant, biocompatible, and suitable mechanical properties.

Nature-derived wound-healing materials include *Aloe vera* leaves, Southern African medicinal plants, *Hypericum hookerianum, Hibiscus sabdaripa calyx* extract, *Moringa olei pera* leaf extract, etc.

There are reports that wound monitoring devices help reduce the costs associated with the diagnosis and treatment of chronic wounds. Chronic wound indicator biomarkers include blood pressure, temperature, pH, temperature, oxygen, and viral infection.

- The main obstacles to be applied

There are many studies on wound-healing substances, but some side effects are that some chemical crosslinking agents cause cytotoxicity. In addition, anti-inflammatory drugs may have limitations in fundamental treatment as well as side effects when administered for a long time. Currently, topical antibiotics, which are mainly used for wounds on the skin, have the disadvantage of becoming resistant if used for a long time, resulting in poor treatment effects. Recently, “super-bacteria” that have become resistant due to the indiscriminate use of antibiotics have emerged as a social problem, and the U.S. Centers for Disease Control and Prevention (CDC) and major dermatology societies no longer recommend the use of preventive topical antibiotics.

Severe wounds are not easy to treat without antibiotics. However, the American and European dermatological societies do not recommend the use of topical antibiotics for preventive purposes. There are also limitations in perception transformation and insufficient alternative antibiotics.

- Future perspectives

With the outbreak of new dermatitis and viruses along with the Fourth Industrial Revolution, research on film, foam, gauze, and electrospun nanofibers for human compatibility and wound healing will be further promoted, and research on biodegradability, antibacterial properties, and mass production will also be in the spotlight.

Foam, gauze, film, electrospinning products, and natural and chemical-derived substances mentioned in this paper have been studied in the direction of superior biocompatibility, antibacterial properties, and ease of production compared to existing products. In particular, substances derived from fish skin have the advantages of mass production and low cost. These studies are expected to provide basic data for the application of cutting-edge wound-healing materials in the future.

As a method to reduce the production cost of wound-healing materials, there is a method of producing them in an OEM manner in countries with low raw materials or labor. In addition, the production cost of wound treatment materials can be reduced only when a country is selected in consideration of customs duties and import and export policies.

The results of this study may accelerate product development for new applications by providing a cornerstone for advanced wound-healing materials.

## Figures and Tables

**Table 2:** The systematization of articles on foam dressing for wound healing, with bibliographic sources.

Authors	Title	Main Content
Gefen, A., et al.	Mechanical and contact characteristics of foam materials within wound dressings: Theoretical and practical considerations in treatment [29]	Foam materials, wound dressings, mechanical characteristics, and contact characteristics
Chiu-Fang Chen., et al.	A multifunctional polyethylene glycol/triethoxysilane-modified polyurethane foam dressing with high absorbency and antiadhesion properties promotes diabetic wound healing [30]	Polyethylene glycol/triethoxysilane-modified polyurethane foam, high absorbency, antiadhesion, diabetic wound healing, and porous structure
Yu-Chi Wang., et al.	The effects of silver-releasing foam dressings on diabetic foot ulcer healing [31]	Silver-releasing foam dressing, diabetic foot ulcers (DFUs), silver sulfadiazine, and wound healing
Chen, C., et al.	Foam dressing and micropower vacuum dressing promote diabetic foot ulcer wound healing by activating the PI3K/AKT/mTOR pathway in rats [32]	Diabetic foot ulcer wound healing, MVD (micropower vacuum dressing), PI3K, AKT, and mTOR
Xie, F., et al.	Alginate foam gel modified by graphene oxide for wound dressing [33]	Alginate foam gel, graphene oxide (CG), wound dressing, vascular remodeling, and antibacterial properties
Woo, K., et al.	Using patient-reported experiences to inform the use of foam dressings for hard-to-heal wounds: perspectives from a wound care expert panel [34]	Hard-to-heal (chronic) wounds, psychosocial issues, local wound care products, wound-related pain, odor, itch, and excessive wound drainage
Watson, F., et al.	In vitro prevention and inactivation of biofilms using controlled-release iodine foam dressings for wound healing [35]	Controlled-release iodine foam dressings, biofilm, wound healing, P. aeruginosa, and *S. aureus*
Du, C., et al.	Shape memory polymer foams with phenolic acid-based antioxidant and antimicrobial properties for traumatic wound healing [36]	Polymer foams, shape memory, phenolic acid, antimicrobial, and antioxidant

**Table 3:** The systematization of articles on gauze dressings for wound healing, with bibliographic sources.

Authors	Title	Main Content
Liang, Y., et al.	Environmentally friendly polylysine gauze dressing for an innovative antimicrobial approach to infected wound management [43]	Polylysine gauze, environmentally friendly, antimicrobial, minimize wound infections, biodegradable, and biocompatibility
Qian, H., T. Lei, and Y. Hu	Negative pressure wound therapy versus gauze dressings in managing open fracture wound of lower limbs: A meta-analysis of randomized controlled trials [44]	Gauze dressings, meta-analysis, negative pressure wound therapy (NPWT), randomized controlled trials (RCTs), and infections
Liu, W.J., et al.	Comparison of Ba-Hao burn ointment gauze and petrolatum gauze in split graft donor site healing: A randomized, prospective, and self-control study [45]	Bahao burn ointment gauze (BHBO), petrolatum gauze, split graft donor site, self-control study, wound-healing time, and Vancouver Scar Scale (VSS)
Fang, H., et al.	A reusable ionic liquid-grafted antibacterial cotton gauze wound dressing [46]	1-vinyl-3-butylimidazolium bromide, 3-aminopropyltriethoxysilane, glycidyl methacrylate, butyl acrylate, cotton gauze, reusable ionic liquid grafted, wound dressing, and antibacterial
Yang, C., et al.	Chitosan and polyhexamethylene guanidine dual-functionalized cotton gauze as a versatile bandage for the management of chronic wounds [47]	Dual-functionalized cotton gauze, chitosan, polyhexamethylene guanidine, and chronic wounds
Nagarjuna Reddy, V., et al.	Gallocatechin-silver nanoparticles embedded in cotton gauze patches accelerated wound healing in diabetic rats by promoting proliferation and inhibiting apoptosis through the Wnt/β-catenin signaling pathway [48]	Cotton gauze, gallocatechin, silver nanoparticles, diabetic rats, proliferation, and wound healing
Xiao, J., et al.	Freeze-thawing chitosan/ions hydrogel coated gauzes releasing multiple metal ions on demand for improved infected wound healing [49]	Gauzes, freeze-thawing, chitosan, ions hydrogel, multiple metal ions, and infected wound healing

**Table 4:** The systematization of articles on electrospun nanofibers for wound healing, with bibliographic sources.

Authors	Title	Main Content
Han, H.R., et al.	Shape memory and breathable waterproof properties of polyurethane nanowebs [28]	Electrospun nanowebs, shape memory polyurethane (SMPU), DSC, pore diameters, breathable, and waterproof
Bao, X., et al.	Antibacterial and antioxidant films based on HA/Gr/TA fabricated using electrospinning for wound healing [59]	Electrospinning, HA/Gr/TA, antibacterial, antioxidant films, and wound healing
Xu, S., et al.	In situ cell electrospun using a portable handheld electrospinning apparatus for the repair of wound healing in rats [60]	Polyvinyl alcohol (PVA), bone marrow-derived stem cells (BMSCs), in situ cell, wound healing, and handheld electrospinning
Liu, X., et al.	Electrospun medicated nanofibers for wound healing [61]	Nanofibers, extracellular matrix (ECM), electrospun, wound healing, nanostructure, and nanocomposite
Guo, S., et al.	Electrospinning of botanicals for skin wound healing [62]	Electrospinning, botanicals, wound healing, loaded drugs, and large-scale manufacturing industry
Chen, R., et al.	HE@ PCL/PCE Gel-Nanofiber Dressing with Robust Self-Adhesion toward High Wound-Healing Rate via Microfluidic Electrospinning Technology [63]	Hyaluronic acid-polylysine (HE), polycaprolactone/poly(citric acid)-ε-lysine (PCL/PCE), microfluidics electrospinning, gel nanofiber, self-adhesion, antibacterial, and wound dressing
Su, S., et al.	Coaxial and emulsion electrospinning of extracted hyaluronic acid and keratin based nanofibers for wound healing applications [64]	Poly(є-caprolactone)/polyethylene oxide, hyaluronic acid(HA), keratin(KR), coaxial electrospinning, and wound-healing applications

**Table 5:** The systematization of articles on chemical materials for wound healing, with bibliographic sources.

Authors	Title	Main Content
Yongping Liang., et al.	Functional Hydrogels as Wound Dressing to Enhance Wound Healing [6]	Multifunctional hydrogel, bioactive wound dressing, wound healing, tissue engineering, bioactive biomaterials, wound regeneration, skin repair, wound closure, and collagen deposition
Zhao, W., et al.	Microfluidic-based functional materials: new prospects for wound healing and beyond [80]	Functional materials, wound healing, physiological stages, microfluidics, well-tailored internal structures, and integrated functions
Fu, W., et al.	Opportunities and challenges of nanomaterials in wound healing: Advances, mechanisms, and perspectives [81]	Nanomaterials, cytotoxicity, side effect, cell damage or death, wound healing, and mechanisms of action
Gowda, B., et al.	Nanoparticle-based therapeutic approaches for wound healing: a review of the state-of-the-art [82]	Nanoparticle, therapeutic approaches, wound healing, physiology, and mechanism in the role of angiogenesis
Diao, W., et al.	Progress in copper-based materials for wound healing [83]	Chronic wounds, diabetic patients, copper, antibacterial, and anti-inflammatory activities
Kharaziha, M., A. Baidya, and N. Annabi	Rational design of immunomodulatory hydrogels for chronic wound healing [84]	Immunomodulatory hydrogels, chronic wound healing, injury, macrophages switch, and anti-inflammatory phenotype
Huang, X., et al.	Balanced chemical reactivity, antimicrobial properties and biocompatibility of decellularized dermal matrices for wound healing [85]	Chemical reactivity, wound healing, antimicrobial properties, decellularized dermal matrices, and biocompatibility
Cheng, H., et al.	The potential of novel synthesized carbon dots derived resveratrol using one-pot green method in accelerating in vivo wound healing [86]	Carbon dots, porcine acellular dermal matrix (pADM), one-pot green method, in vivo, and wound healing

**Table 7:** The systematization of articles on monitoring wound healing, with bibliographic sources.

Authors	Title	Main Content	Bibliographic Source
Zhang, Y., et al.	An integrated smart sensor dressing for real-time wound microenvironment monitoring and promoting angiogenesis and wound healing	Smart sensor dressing, real-time wound microenvironment monitoring, wound healing, and angiogenesis	[16]
Sol Youn., et al.	Biomimetic Materials for Skin Tissue Regeneration and Electronic Skin	Biomimetic, wound healing, wound dressing, E-skin, smart bandage, nature inspired, real-time monitoring, nanostructure, and diatom	[142]
Zhaosu Wang., et al.	Hydrogel-Based Energy Harvesters and Self-Powered Sensors for Wearable Applications	Hydrogel, triboelectric, piezoelectric, thermoelectric, wearable, energy harvester, self-powered, and sensor	[143]
Hanning Wu., et al.	Development and Prospective Applications of 3D Membranes as a Sensor for Monitoring and Inducing Tissue Regeneration	Tissue regeneration, 3D printing, sensors, biomaterials, and artificial membrane application	[144]
Shanshan Jin., et al.	Progress of Hydrogel Dressings with Wound Monitoring and Treatment Functions	Wound monitoring, treatment, hydrogel, and wound dressing	[145]
Lirong Wang., et al.	Multifunctional hydrogel as wound dressing for intelligent wound monitoring	Intelligent wound monitoring, multifunctional hydrogel, wound Ph, and machine learning technology.	[17]
